# ViTax-RAG: a retrieval-augmented language modeling tool for viral contig taxonomic classification

**DOI:** 10.1093/bioadv/vbag247

**Published:** 2026-08-24

**Authors:** Feng Zhou, Lan Cao, Yushuang He, Jiaxing Bai, Ying Wang

**Affiliations:** Department of Automation, National Institute for Data Science in Health and Medicine, State Key Laboratory of Mariculture Breeding, Xiamen Key Laboratory of Big Data Intelligent Analysis and Decision, Xiamen University, Xiamen, Fujian 361102, China; Department of Automation, National Institute for Data Science in Health and Medicine, State Key Laboratory of Mariculture Breeding, Xiamen Key Laboratory of Big Data Intelligent Analysis and Decision, Xiamen University, Xiamen, Fujian 361102, China; Department of Automation, National Institute for Data Science in Health and Medicine, State Key Laboratory of Mariculture Breeding, Xiamen Key Laboratory of Big Data Intelligent Analysis and Decision, Xiamen University, Xiamen, Fujian 361102, China; Department of Automation, National Institute for Data Science in Health and Medicine, State Key Laboratory of Mariculture Breeding, Xiamen Key Laboratory of Big Data Intelligent Analysis and Decision, Xiamen University, Xiamen, Fujian 361102, China; Department of Automation, National Institute for Data Science in Health and Medicine, State Key Laboratory of Mariculture Breeding, Xiamen Key Laboratory of Big Data Intelligent Analysis and Decision, Xiamen University, Xiamen, Fujian 361102, China

## Abstract

**Motivation:**

Taxonomic classification of viral metagenomic contigs remains difficult for short or divergent sequences. Reference-based methods are precise when close homologs exist, whereas representation-based models can generalize beyond direct matches but lack explicit biological evidence.

**Results:**

Here, we present ViTax-RAG, a retrieval-augmented framework that integrates alignment-derived evidence with learned sequence representations for robust viral classification. ViTax-RAG reformulates BLAST as a domain-specific retrieval module and integrates retrieved homology information into a sequence modeling framework, thereby enabling the complementary use of alignment-based and representation-based signals. We evaluated ViTax-RAG on in-distribution (ID) and within-genus out-of-distribution (OOD) datasets, where it consistently outperformed current viral taxonomy methods at comparable taxonomic endpoints and supported fragment lengths. The pipeline processed all 195 728 GOV 2.0 contigs; 87.2% of predictions terminated at class, demonstrating hierarchical backoff rather than fine-rank accuracy on data without ground truth.

**Availability and implementation:**

ViTax-RAG is implemented in Python and is freely available at GitHub (https://github.com/Ying-Lab/ViTax-Rag) under an open-source license. Documentation and example workflows are provided to facilitate integration into metagenomic analysis pipelines.

## 1 Introduction

Metagenomic sequencing has greatly expanded the discovery of viruses across diverse environments (Paez-Espino *et al.* 2016). Taxonomic classification of viral sequences is therefore essential for characterizing viral diversity, exploring host-virus interactions, and monitoring emerging pathogens. The rapid growth of metagenomic datasets has generated large numbers of viral contigs, creating a demand for automated and scalable computational tools for taxonomic assignment (Roux *et al.* 2019). However, reliable classification remains challenging, particularly for short contigs, fragmented assemblies, and highly divergent sequences that lack close homologs in current reference databases.

Existing computational approaches generally fall into two broad categories: database-based and machine-learning methods (Tian *et al.* 2024). Reference-based methods, such as Kraken2 (Wood *et al.* 2019) and CAT (von Meijenfeldt *et al.* 2019), rely on explicit sequence matching or k-mer-based similarity against curated databases. These tools typically achieve high precision when closely related references are available, but their performance deteriorates for short sequences, incomplete genomes, or taxa absent from the reference database. Alternatively, representation-learning approaches based on genomic language models have recently emerged. In our previous work, we developed ViTax (He *et al.* 2025), a language-model-based framework that learns contextual sequence embeddings and demonstrates improved generalization to divergent viral sequences. Nevertheless, these methods often lack explicit biological evidence grounding, which may limit interpretability and reduce confidence in hierarchical taxonomic assignment.

Recent advances in natural language processing have demonstrated the effectiveness of retrieval-augmented generation (RAG) (Lewis *et al.* 2020), where external evidence is dynamically retrieved and integrated with neural representations to enhance robustness and reliability. Inspired by this paradigm, we observe that alignment-based homology search in microbial genomics can be naturally reframed as a domain-specific retrieval mechanism. In particular, sequence alignment tools such as BLAST (Altschul *et al.* 1990, 1997, Johnson *et al.* 2008, Camacho *et al.* 2009) inherently retrieve homologous sequences that provide biologically meaningful external evidence, yet they are rarely integrated in a unified framework with modern language models.

Here, we present ViTax-RAG, a user-friendly Python package for reference-backed viral contig taxonomy classification. ViTax-RAG uses BLASTn as a domain-specific retriever, ranks candidate homologs with an interpretable multi-feature score, integrates the selected homologous sequence evidence with the query, encodes the resulting sequence with a bidirectional Hyena (Bi-Hyena) model, and performs confidence-aware taxonomy-tree voting. The explicit retrieval and taxonomy-tree stages make the evidence source traceable, permit reference-database updates without retraining the encoder, and return a taxonomic rank and confidence rather than a free-form generated label. On the fixed 1515-genome test set, ViTax-RAG achieved 97.7% family F1 and 94.5% genus F1, outperforming current viral taxonomy tools at comparable endpoints: geNomad, PhaGCN2.0, PhaGCN_Cluster, and vConTACT3 at family rank, and PhaGCN_Cluster and vConTACT3 at genus rank. Across controlled 500–4000-bp fragments, ViTax-RAG achieved the highest established-genus accuracy in all supported comparisons with Kraken2, CAT, geNomad, PhaGCN_Cluster, and vConTACT3, with its clearest advantages on short contigs. On the within-genus distribution-shift benchmark, ViTax-RAG again outperformed the evaluated methods across supported fragment lengths, while genus F1 increased from 50.6% at 500 bp to 73.8% at 4000 bp.

## 2 Methods

**Figure 1:**
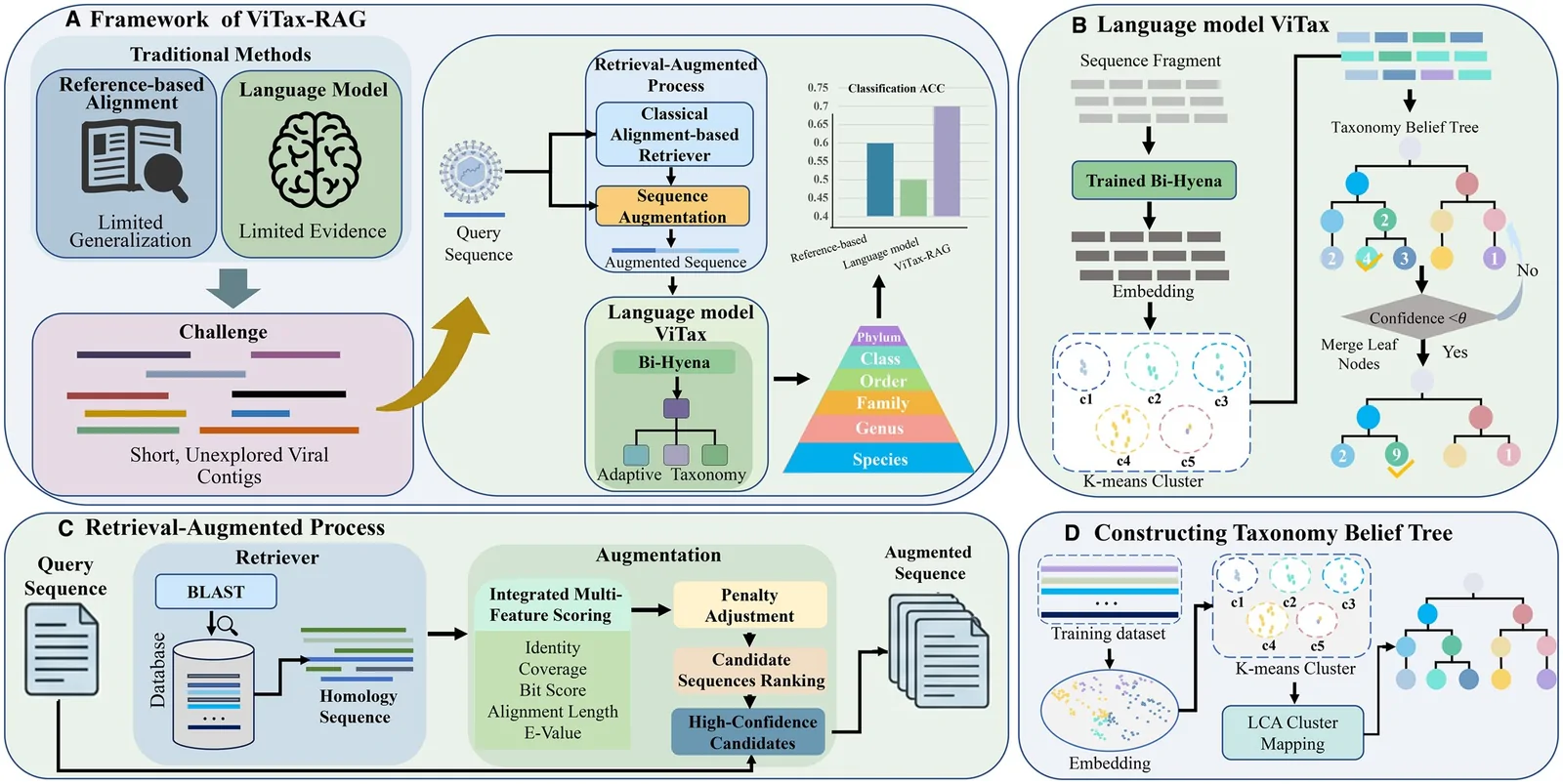
Overview of the ViTax-RAG framework. (A) Overall framework integrating BLASTn retrieval, sequence augmentation, a Bi-Hyena encoder, *K*-means cluster assignment, and taxonomy-tree voting; (B) Bi-Hyena encoder and taxonomy belief tree, which map sequence windows to taxonomic evidence; (C) Retrieval process, where BLASTn reports homologous subjects, candidates are ranked by the multi-feature homology score, and the top-ranked homologous sequence evidence is appended to the query; (D) Construction of the taxonomy belief tree using embedding-based clustering and LCA mapping.

The overall framework of ViTax-RAG is illustrated in Fig. 1A. The nomenclature distinguishes three stages of the model: ViTax is the published baseline with a unidirectional Hyena encoder, ViTax-BiHyena is the bidirectional sequence-only variant, and ViTax-RAG adds an explicit retrieval step before the otherwise unchanged Bi-Hyena/K-means/taxonomy-tree classification path. Unlike natural-language RAG systems, ViTax-RAG does not generate free-form labels. It retrieves nucleotide evidence from a fixed reference database, augments the query when a hit is available, and assigns labels through a curated taxonomy belief tree. When no non-root taxonomic path meets the confidence threshold, ViTax-RAG reports unclassified rather than proposing a novel taxon.

The dataset was constructed from the 2023 NCBI Virus RefSeq collection and comprised 3977 complete dsDNA viral genomes with usable genus labels (2462 training and 1515 test genomes). Genome eligibility and the 6:4 within-genus split were defined using the RefSeq records and taxonomy available at dataset construction. Only genera represented by at least two genomes were retained; splitting preceded 2000-bp/400-bp fragmentation and retained at least one test genome for small genera, preventing fragments from the same genome from crossing partitions. ICTV VMR MSL39 v1 was applied subsequently to harmonize current names for reporting. MSL39 reconciliation yielded current VMR rows for 2448 training and 1505 test genomes; unmatched accessions remained in the fixed partitions but were excluded from VMR-dependent metrics. RefSeq was selected as a curated collection with more standardized sequence and taxonomy records than unrestricted GenBank, while complete genomes provide consistent context for training, controlled fragmentation, and retrieval-database construction.

The BLAST database contains exactly the 2462 training-genome FASTA records identified by RefSeq accessions; the fixed 1515-genome test set is used only as query input and is absent from the retrieval database. Taxonomic reporting is versioned with ICTV VMR MSL39 v1 (file release dated 12 September 2024), and rank-specific metrics require a VMR label at the evaluated rank.

Caudoviricetes account for 1803/2462 training genomes (73.2%), so pooled results are supplemented by class-stratified analyses. The VMR-matched training set contained 632 current genera, and 623 of the 629 current test genera were also represented in training. Because species were predominantly singletons, genus was used as the principal fine-rank endpoint. Dataset composition and current taxonomic breadth are summarized in Supplementary Tables S1 and S2, available at supplementary material  *Bioinformatics Advances* online; complete taxonomic distributions and per-species/per-genus counts are provided in the Supplementary Data, available at supplementary material  *Bioinformatics Advances* online.

### 2.1 Retrieval-augmented framework

To enhance the model’s ability to use local homology evidence, each query sequence is searched against the local BLASTn database (Fig. 1C). Retrieval used NCBI BLAST + v2.12.0 (Camacho *et al.* 2009), program blastn, task megablast, E-value threshold 10, both query strands, and at most 500 target sequences. All hits satisfying this command-level E-value threshold were ranked without additional identity, query-coverage, alignment-length, bit-score, or other post-BLAST filters. A query was retrieval-negative only when BLAST returned no output row. Tabular output fields were qseqid sseqid sstart send pident length mismatch gapopen bitscore evalue qcovs.

Candidate homologs were ranked by a unified multi-feature score. Identity and query coverage were scaled to fractions, whereas bit score, transformed E-value significance, and alignment length were maximum-normalized within the hits returned for each individual query. The candidate set therefore contains at most 500 hits from one query and never includes hits from other queries or from an encoder/inference batch. Complete normalization formulas and the handling of singleton candidate sets, tied values, zero denominators, and empty candidate sets are provided in Supplementary Methods (“Candidate-score normalization and ranking”). The normalized E-value significance (${\tilde{E}}_{i}$), identity (${\tilde{I}}_{i}$), query coverage (${\tilde{C}}_{i}$), and bit score (${\tilde{B}}_{i}$) are combined as


(1) $$S_{i}={\tilde{E}}_{i}^{\alpha }{\tilde{I}}_{i}^{\beta }{\tilde{C}}_{i}^{\gamma }{\tilde{B}}_{i}^{\delta }.$$

The fixed exponents are α=0.4, β=0.6, γ=0.5, and δ=0.5. Normalized alignment length ${\tilde{L}}_{i}$ is incorporated with exponent η=0.3, and gap openings and mismatches are penalized to give


(2) $$S_{i}^{*}=S_{i}{\tilde{L}}_{i}^{\eta }{\left(1+g_{i}\right)}^{-1}\;\text{exp}(-2m_{i}/L_{i}),$$

where $g_{i}$, $m_{i}$, and $L_{i}$ denote gap openings, mismatches, and aligned bases. The multiplicative form produces a “short-board” effect: weak performance in any key homology dimension lowers the complete score rather than allowing one strong statistic to dominate. Candidates are ranked by $S_{i}^{*}$. Scores within a relative difference of 0.5% are treated as near-ties and resolved deterministically by higher identity, higher coverage, fewer gaps, fewer mismatches, longer alignment, and stable reference order.

The scoring exponents, penalty constants, and tie tolerance were selected on a held-out development split drawn only from the training partition and fixed before the final ICTV, OOD, or GOV evaluations. Candidate-ranking consistency and downstream taxonomic performance on this development split guided selection; no final test or OOD data were used. The candidate with the highest composite score $S_{i}^{*}$ is selected, its subject sequence is loaded, and the complete substring subject[send:] in stored FASTA orientation is concatenated to the original query. The augmented length is not capped. Consequently, there is no query-length-specific branch: a hit-positive query at or above 4 kb follows the same scoring, suffix-concatenation, and windowing procedure and may become longer, whereas a no-hit query remains unchanged and proceeds through the same Bi-Hyena encoder, *K*-means index, and taxonomy tree. Operationally, this is the ViTax-BiHyena retrieval-off path, not the original ViTax model.

### 2.2 Adaptive taxonomy classification

The final adaptive taxonomy classification stage (Fig. 1B) assigns viral contigs to the lowest taxonomic rank supported by sufficient confidence. Windowing follows the released historical split_string4 rule. For a sequence of length L ≥ 2000 bp, complete 2000-bp windows begin at offsets 0,400,800,… while the start plus 2000 does not exceed *L*; an additional right-aligned terminal window is not added. For L  ≥  2000 bp, the complete sequence is retained once as a single short window. Thus a retrieval-negative query of 500, 1000, or 1500 bp contributes one short window on the forward strand and one on the reverse-complement strand when bidirectional inference is enabled. Nucleotides are tokenized one character per token with special-token insertion disabled. Every window is encoded by the trained Bi-Hyena model and assigned to a pre-learned K-means cluster. Inference uses PyTorch evaluation mode with a fixed seed 0, disabling the 0.1 embedding-dropout layer used during training. Each cluster contributes votes to nodes in the taxonomy belief tree. The reported confidence is the normalized support for the selected node across all forward and reverse-complement windows. The default confidence threshold is θ=0.6. We selected θ=0.6 on the same held-out development split used for the retrieval parameters to balance the terminal genus fraction against erroneous low-confidence assignments and excessive upward backoff, and locked it before all final test and OOD evaluations. A genus label is therefore not forced. If the strongest genus-level support is below θ, the tree merges evidence upward and re-evaluates support at family, order, or class; if no non-root path is sufficiently supported, the output is ‘unclassified’. Hierarchical backoff and abstention reduce forced fine-rank assignment for divergent inputs. The output is therefore either ‘unclassified’ or a label of the form TaxonName_TaxonLevel.

Rank-specific metrics were calculated only for inputs with VMR truth at the evaluated rank. Predictions terminating above that rank, as well as unclassified outputs, were counted as false negatives for recall but excluded from the precision denominator; deeper predictions were projected to their lineage ancestor. In Table 1, $N_{\text{assigned}}$ denotes truth-evaluable inputs with a prediction at the evaluated rank, and lineage coverage is $N_{\mathrm{assigned}}/1515$. Exact-label matches were counted as true positives (*TP*); precision is $TP/N_{\text{assigned}}$, recall is $TP/N_{\text{truth}}$, and F1 is their harmonic mean.

**Table 1:** Rank-aware performance on the fixed 1515-genome test set.

Method	Rank	$N_{\text{truth}}$	$N_{\text{assigned}}$	Lineage coverage (%)	Precision (%)	Recall (%)	F1 (%)
ViTax-RAG	Phylum	1346	1320	87.1	99.8	97.9	98.9
ViTax-RAG	Class	1387	1359	89.7	99.8	97.8	98.8
ViTax-RAG	Order	282	258	17.0	99.2	90.8	94.8
ViTax-RAG	Family	855	824	54.4	99.5	95.9	97.7
ViTax-RAG	Genus	1505	1402	92.5	97.9	91.2	94.5
ViTax	Phylum	1346	1310	86.5	99.8	97.2	98.5
ViTax	Class	1387	1350	89.1	99.8	97.1	98.4
ViTax	Order	282	248	16.4	99.6	87.6	93.2
ViTax	Family	855	811	53.5	99.6	94.5	97.0
ViTax	Genus	1505	1372	90.6	97.9	89.2	93.4
geNomad v1.12.0	Phylum	1346	1339	88.4	99.8	99.3	99.5
geNomad v1.12.0	Class	1387	1375	90.8	99.9	99.0	99.4
geNomad v1.12.0	Order	282	264	17.4	100.0	93.6	96.7
geNomad v1.12.0	Family	855	693	45.7	99.4	80.6	89.0
geNomad v1.12.0	Genus	1505	–	–	–	–	–
PhaGCN2.0 v2.3	Family	855	672	44.4	98.5	77.4	86.7
PhaGCN_Cluster	Phylum	1346	1228	81.1	97.0	88.5	92.5
PhaGCN_Cluster	Class	1387	1260	83.2	97.1	88.2	92.4
PhaGCN_Cluster	Order	282	245	16.2	98.8	85.8	91.8
PhaGCN_Cluster	Family	855	672	44.4	99.1	77.9	87.2
PhaGCN_Cluster	Genus	1505	230	15.2	66.1	10.1	17.5
vConTACT3 v3.1.6	Phylum	1346	1333	88.0	85.0	84.2	84.6
vConTACT3 v3.1.6	Class	1387	1373	90.6	82.5	81.7	82.1
vConTACT3 v3.1.6	Order	282	269	17.8	11.9	11.3	11.6
vConTACT3 v3.1.6	Family	855	789	52.1	60.8	56.1	58.4
vConTACT3 v3.1.6	Genus	1505	1439	95.0	62.9	60.1	61.5

*Note.* $N_{\text{truth}}$ is calculated independently at each rank from populated ICTV VMR MSL39 v1 fields; missing ancestral ranks are not imputed.

Values are percentages except *N*; unsupported ranks are dashes. The deepest-rank distribution is in Table S3, available at supplementary material  *Bioinformatics Advances* online. PhaGCN_Cluster and vConTACT3 complete-set results are descriptive because their databases contain test accessions.

For the length-stratified ablation, genus accuracy at length *L* is the number of contigs assigned the correct genus divided by all test contigs at that length. The retrieval-only effect was estimated by disabling augmentation in the matched ViTax-BiHyena control (—augment false) while holding the Bi-Hyena checkpoint and encoder configuration, K-means model, taxonomy tree and index, windowing, and confidence threshold fixed. The original ViTax result was retained only as a historical baseline. BLAST-only top-hit label transfer emits the genus lineage of the first-ranked training subject without computing an LCA across hits.

### 2.3 Constructing taxonomy belief tree

ViTax-RAG constructs a taxonomy belief tree to link the learned genomic embedding space to the established biological taxonomy hierarchy (Fig. 1D). The process begins by generating fragment-level embeddings for all training genomes using the Bi-Hyena model, followed by *K*-means clustering to partition the embedding space into coherent regions. Each resulting cluster is assigned a definitive taxonomy label by computing the lowest common ancestor (LCA) of all member sequences based on the viral evolutionary tree. The LCA-annotated clusters are then organized into the taxonomy belief tree. The resulting non-parametric tree encodes both embedding similarity and biological taxonomy for adaptive classification during inference.

## 3 Datasets and benchmark design

We evaluated ViTax-RAG and the controls defined above using three complementary settings. First, formal metrics use the 2462-genome training partition for the BLAST database and taxonomy belief tree and the same fixed 1515-genome test set for evaluation. The extensive train–test taxonomic overlap makes this primarily a reference-proximal known-taxon evaluation rather than evidence for unconstrained novel-taxon discovery.

Second, to test short-contig behavior on this reference split, held-out genomes were fragmented in silico at 500, 1000, 1500, 2000, 3000, and 4000 bp. The length-stratified ablation evaluates every contig in the corresponding fragment manifest; a separate accession-balanced subset is used for rank-aware and terminal-rank diagnostics. Clean fragmentation does not reproduce all artifacts of real assembly, but it isolates the effects of sequence length and retrieval.

Third, the OOD benchmark uses an OOD-specific checkpoint, K-means index, and taxonomy tree. The split contains 1466 training and 494 test accessions (1960 in total), with all 143 genera represented in both subsets. Split construction used Dashing v1.0.2-4-g0635 with the containment index, k=21, and the default HyperLogLog sketch size of 1024 bytes. The containment index estimates the directional fraction of one genome’s 21-mer set contained in another genome’s 21-mer set. Genomes whose row-mean containment index against other members of the same genus exceeded 0.3 were removed to reduce highly redundant representatives. Two-cluster spectral clustering was then applied to the remaining within-genus containment matrix (precomputed affinity; random seed 42), assigning separated genome clusters to training and test. The benchmark therefore evaluates within-genus distribution shift rather than unseen-genus recognition. Because 0.3 is a cutoff on mean directional containment and depends on genome length and averaging across multiple genomes, it is neither a Jaccard/identity threshold nor uniquely convertible to ANI. A separate BLAST database was constructed exclusively from the 1466 OOD training genomes, comprising 73 997 559 bp in total. Accession overlap, canonical forward/reverse-complement exact-sequence overlap, and test-accession top self-hits were all zero. Test genomes were divided into non-overlapping fragments at 500, 1000, 1500, 2000, 3000, and 4000 bp; a genome shorter than the nominal length was retained once as its complete sequence, so every length covers all 494 accessions. OOD inference uses the candidate-scoring and sequence-augmentation rules described above.

We additionally ran the current PhaGCN_Cluster and vConTACT3 releases directly on these OOD test-fragment FASTA files. PhaGCN_Cluster was evaluated at 2000, 3000, and 4000 bp; 500–1500-bp inputs fall below its documented default 1700-bp minimum and are reported as unsupported rather than forced through a modified threshold. vConTACT3 was evaluated at all six lengths. Both tools used their current bundled/official databases rather than databases constructed from the OOD training genomes: exact-accession audit found 435/494 (88.1%) OOD test accessions in the PhaGCN_Cluster database and 332/494 (67.2%) in vConTACT3 v230. We therefore report both all-fragment results and method-specific accession-overlap-free subsets (59 PhaGCN-clean and 162 vConTACT3-clean accessions). The endpoint is exact recovery of the established ICTV genus; a candidate novel cluster is retained as an output but is not treated as a correct known-genus label.

Current virome comparisons used geNomad v1.12.0 (Camargo *et al.* 2024) with database v1.9, PhaGCN_Cluster (Xia *et al.* 2026) from official commit 8da942a with its bundled MSL39 database, and vConTACT3 v3.1.6 (Bolduc *et al.* 2025) with the checksum-verified v230 database on the same fixed 1515-genome test set. PhaGCN2.0 v2.3 (Shang *et al.* 2021) provides a supporting family-rank comparison. The downloaded PhaGCN_Cluster and vConTACT3 databases contain exact accessions for 1079/1515 (71.2%) and 1124/1515 (74.2%) test genomes, respectively (Table S10, available at supplementary material  *Bioinformatics Advances* online). We therefore report both complete-set results and database-specific accession-overlap-free subsets (436 and 391 genomes), and do not interpret complete-set results as independent generalization estimates. Unsupported ranks are shown as dashes rather than scored as zero. Kraken2 v2.1.3 and CAT_pack v6.0 are commonly used taxonomic classification methods in metagenomic analysis, including viral sequence classification, and are retained as training-set-matched comparisons using databases constructed from the corresponding ViTax-RAG training genomes.

## 4 Performance evaluation

### 4.1 Rank-aware performance on the full held-out test set

Rank-specific metrics are reported because a correct coarse ancestor and a correct genus call provide different levels of information. Table 1 gives precision, recall, F1, and lineage assignment coverage at every supported rank. Table 2 summarizes fragment-level established-genus accuracy on the RefSeq and within-genus OOD sequence sets. ViTax-RAG achieved 97.9% genus precision, 91.2% recall, and 94.5% F1, with 93.2% of inputs terminating at genus. The original ViTax baseline achieved 93.4% genus F1, 1.1 percentage points below ViTax-RAG.

**Table 2:** Established-genus accuracy by fragment length on the RefSeq (A) and within-genus OOD (B) sets.

A. RefSeq dataset							
Length	ViTax-RAG	ViTax	Kraken2	CAT	geNomad	PhaGCN_Cluster	vConTACT3
500 bp	0.809	0.008	0.800	0.747	0.162360	–	0.000029
1000 bp	0.868	0.256	0.843	0.793	0.215813	–	0.000154
1500 bp	0.920	0.849	0.864	0.812	0.238337	–	0.000203
2000 bp	0.932	0.899	0.877	0.824	0.254662	0.041186	0.000350
3000 bp	0.939	0.912	0.893	0.841	0.267645	0.058015	0.000998
4000 bp	0.947	0.927	0.905	0.850	0.277235	0.066806	0.001734

B. Within-genus OOD dataset						
Length	ViTax-RAG	Kraken2	CAT	geNomad	PhaGCN_Cluster	vConTACT3
500 bp	0.633	0.500	0.502	0.170892	–	0.000000
1000 bp	0.647	0.557	0.527	0.228155	–	0.000058
1500 bp	0.655	0.585	0.545	0.250022	–	0.000175
2000 bp	0.666	0.605	0.556	0.266589	0.058104	0.000234
3000 bp	0.649	0.628	0.569	0.282192	0.079010	0.000884
4000 bp	0.673	0.646	0.574	0.292131	0.085328	0.002489

Panel A retains the historical ViTax baseline. OOD Kraken2 uses the OOD-training-only database; other external tools use current databases. vConTACT3 reports candidate-set hit accuracy, not the exact-single-label P/R/F1 in Table 1.

geNomad achieved 99.5%, 99.4%, and 96.7% F1 at phylum, class, and order, and 89.0% F1 at family; no comparable genus endpoint was available. PhaGCN2.0 was compared only at family because its reference label space is primarily family-level; version 2.3 provides genus endpoints only for a limited subset of taxa lacking an ICTV family assignment, rather than a general genus endpoint comparable across the 629 truth genera. Its supporting family F1 was 86.7%. ViTax-RAG family F1 was 97.7%, exceeding PhaGCN2.0, PhaGCN_Cluster, and vConTACT3 by 11.0, 10.5, and 39.3 percentage points, respectively. Among the 1505 inputs with genus truth from 629 distinct genera, ViTax-RAG assigned 1402 and correctly classified 1373, recovering 565 distinct truth genera. PhaGCN_Cluster assigned 230, correctly classified 152, and recovered 20 distinct truth genera (genus F1 17.5%). vConTACT3 assigned 1439 and correctly classified 905, recovering 439 distinct truth genera (genus F1 61.5%). The exact-label counts measure recovery of established ICTV genera.

On a common 155-genome subset with no exact accession in either external database, ViTax-RAG family/genus F1 was 96.5%/87.6%, compared with 40.4%/unsupported for geNomad, 7.8%/0% for PhaGCN_Cluster, and 1.7%/2.6% for vConTACT3. Database-specific overlap-free results were likewise 6.0%/0% for PhaGCN_Cluster and 4.6%/2.6% for vConTACT3 (Table S11, available at supplementary material  *Bioinformatics Advances* online). Across these overlap-free comparisons, ViTax-RAG recovered established family and genus labels more accurately than the current tools.

Because 73.2% of training genomes are Caudoviricetes, pooled genus results were also stratified. ViTax-RAG genus F1 was 98.1% for Caudoviricetes and 80.6% for all other assigned classes combined. Among classes with at least 20 final-test genomes, ViTax-RAG F1 ranged from 63.3% for Papovaviricetes to 100% for Pokkesviricetes; full counts and per-class metrics are provided in Supplementary Tables S6–S8, available at supplementary material  *Bioinformatics Advances* online. The 17.5-point F1 gap limits extrapolation to under-represented viral classes.

### 4.2 Main ablation and retrieval stratification

Table 3 reports the length-stratified ID ablation and OOD retrieval attribution. ViTax-RAG achieved 0.809–0.947 exact-genus accuracy across 500–4000 bp. At 500 bp, BLAST-only top-hit label transfer was 0.008 higher than ViTax-RAG (0.817 versus 0.809); the BLAST hit rate was 0.847, and the top-hit genus was correct for 96.38% of hit-positive contigs. From 1000 bp onward, ViTax-RAG exceeded the sequence-free BLAST-only control by 0.016, 0.049, 0.049, 0.042, and 0.039, respectively. Both sequence-only baselines improved with sequence length, while the matched ViTax-BiHyena ablation showed that retrieval contributed most strongly at 500–1000 bp.

**Table 3:** Retrieval attribution by fragment length.

A. ID ablation accuracy						
Length	*N*	ViTax-RAG	ViTax-BiHyena	ViTax	BLAST-only top-hit label transfer	BLAST hit rate
500 bp	2 08 031	0.809	0.008	0.008	**0.817**	0.847
1000 bp	1 03 650	**0.868**	0.271	0.256	0.852	0.882
1500 bp	68 831	**0.920**	0.854	0.849	0.871	0.899
2000 bp	51 425	**0.932**	0.900	0.899	0.883	0.911
3000 bp	34 060	**0.939**	0.916	0.912	0.897	0.924
4000 bp	25 372	**0.947**	0.933	0.927	0.908	0.932

B. OOD retrieval stratification						
Length	$N_{\text{hit}}$	$N_{\text{no}0\text{hit}}$	BLAST hit rate	BLAST-only accuracy	ViTax-RAG accuracy, hit	ViTax-RAG accuracy, no hit
500 bp	37 555	31 588	0.5431	0.5077	0.8836	0.3343
1000 bp	20 706	13 753	0.6009	0.5605	0.8802	0.2955
1500 bp	14 426	8456	0.6305	0.5890	0.8774	0.2760
2000 bp	11 120	5970	0.6507	0.6080	0.8774	0.2732
3000 bp	7651	3664	0.6762	0.6331	0.8370	0.2555
4000 bp	5843	2595	0.6925	0.6492	0.8684	0.2328

Panel A reports exact-genus accuracy on all ID contigs. Panel B reports OOD BLAST hit rate, all-contig BLAST-only accuracy, and ViTax-RAG accuracy within hit/no-hit strata. Unclassified and coarse-rank outputs are incorrect; each stratum uses all its contigs as the denominator. Bold values indicate the best performance for each metric.

The OOD BLAST hit rate increased from 0.5431 at 500 bp to 0.6925 at 4000 bp, and BLAST-only top-hit accuracy increased from 0.5077 to 0.6492. ViTax-RAG accuracy was consistently higher for hit-positive contigs (0.8370–0.8836) than for no-hit contigs; no-hit accuracy declined from 0.3343 at 500 bp to 0.2328 at 4000 bp. Thus, the retrieval-negative sequence-only path retained limited but non-zero fallback capability, but its performance remained substantially lower than that of the hit-positive stratum. The count-weighted averages of the two strata are the all-fragment ViTax-RAG accuracies reported in Table 2, Panel B.

A separate accession-balanced analysis reports rank-aware genus precision, recall, F1, and terminal-rank distributions (Supplementary Tables S4 and S5, available at supplementary material  *Bioinformatics Advances* online). At 500 bp, ViTax-RAG terminated at genus for 79.2% of inputs and achieved genus precision, recall, and F1 of 94.5%, 74.9%, and 83.6%; ViTax-BiHyena terminated at genus for 5.9% and achieved 2.2%, 0.1%, and 0.3%. Only 7.7% of ViTax-RAG outputs terminated at class and 0.1% at family, and neither group received genus-level credit. Across 500, 1000, 2000, and 4000 bp, ViTax-RAG versus ViTax-BiHyena genus F1 was 83.6% versus 0.3%, 87.1% versus 16.7%, 91.2% versus 86.1%, and 93.6% versus 90.1%, respectively. Complete length-specific terminal-rank distributions are provided in Table S5, available at supplementary material  *Bioinformatics Advances* online.

### 4.3 OOD evaluation under within-genus distribution shift

Under this within-genus shift, rank-aware genus F1 and all-fragment exact-genus accuracy use the different denominators defined in Methods. ViTax-RAG genus F1 increased from 50.6% at 500 bp to 73.8% at 4000 bp, compared with 1.0% and 62.6% for ViTax-BiHyena. ViTax-RAG therefore exceeded the matched ViTax-BiHyena control at both reported lengths, isolating the contribution of retrieval.

Under the all-fragment exact-genus endpoint, ViTax-RAG accuracies at 500, 1000, 1500, 2000, 3000, and 4000 bp were 0.633, 0.647, 0.655, 0.666, 0.649, and 0.673, respectively (Table 2, Panel B). The corresponding Kraken2 values were 0.500, 0.557, 0.585, 0.605, 0.628, and 0.646, and the CAT values were 0.502, 0.527, 0.545, 0.556, 0.569, and 0.574. ViTax-RAG therefore remained more accurate than both training-set-matched baselines across all six fragment lengths.

We also evaluated current database releases on the same fragments. Exact established-genus recovery was lower for both protein/gene-sharing tools (Table S9, available at supplementary material  *Bioinformatics Advances* online). PhaGCN_Cluster genus F1 was 10.8%, 14.4%, and 15.3% at 2000, 3000, and 4000 bp, respectively, versus 69.8%–73.8% for ViTax-RAG. vConTACT3 exact-genus F1 was 0%–0.27% across 500–4000 bp because most fine-rank outputs were candidate clusters rather than the established truth label. On method-specific accession-overlap-free subsets, ViTax-RAG/PhaGCN_Cluster F1 was 36.4%/2.6%, 38.1%/3.3%, and 39.2%/3.3% at 2000, 3000, and 4000 bp; ViTax-RAG/vConTACT3 F1 ranged from 17.3% to 52.2% versus 0%–0.05%. Across the accession-overlap-free subsets, ViTax-RAG recovered established genera more accurately than either protein/gene-sharing tool.

### 4.4 GOV 2.0 large-scale feasibility and terminal-rank demonstration

The Global Ocean Viromes 2.0 (GOV 2.0) dataset (Gregory *et al.* 2019) was used as a real-data terminal-rank and computational audit, not an accuracy benchmark, because complete ground-truth taxonomy is unavailable. The official at-least-10-kb/circular dataset contains 1 95 728 contigs (median 16.2 kb; range 10–505 kb): 1 25 308 at 10–20 kb, 35 641 at 20–30 kb, 25 135 at 30–50 kb, and 9644 above 50 kb (Table S12, available at supplementary material  *Bioinformatics Advances* online). The dataset therefore does not directly test the 500 bp–4 kb regime; short-input performance is quantified separately by the controlled RefSeq and OOD fragment experiments (Table 2 and Supplementary Tables S4 and S5, available at supplementary material  *Bioinformatics Advances* online). No independent, accessible dataset of ground-truthed 500-bp–4-kb viral contigs assembled from real samples was available for this study; validation on real assembled short contigs therefore remains future work.

Across all 1 95 728 GOV 2.0 contigs, ViTax-RAG left 1475 (0.754%) unclassified, while 1 70 676 (87.201%) terminated at class, 2956 (1.510%) at order, 16 634 (8.499%) at family, and 3987 (2.037%) at genus (Table S13, available at supplementary material  *Bioinformatics Advances* online). Most GOV contigs therefore received a class-level terminal label, illustrating confidence-aware backoff when fine-rank support was insufficient. Processing all 1 95 728 contigs confirmed end-to-end execution on a large virome dataset, but this single run was not a throughput-scaling experiment. Figure S1, available at supplementary material  *Bioinformatics Advances* online visualizes the resulting taxonomic composition.

Ecological use of ViTax-RAG requires the taxonomic output to be combined with appropriate upstream and quantitative processing: contigs should be dereplicated, binned, or clustered into viral populations/vOTUs, reads should be mapped back to estimate normalized abundance, and abundance should then be aggregated only at the supported terminal ranks. Raw contig counts are not richness estimates, and an invariant class-only label contributes little to beta-diversity analysis. Conversely, supported family/genus assignments can provide a taxon-resolved annotation layer for abundance-aware community profiling. Without sample-level abundance data, the GOV outputs document taxonomic resolution and backoff rather than ecological performance.

## 5 Implementation

ViTax-RAG is implemented in Python and provides installed-package and portable command-line interfaces for end-to-end inference on Linux. The software supports automatic device selection (—device auto) for CPU or GPU execution and allows users to configure confidence thresholds, sequence chunking, batch size, reverse-complement inference, and retrieval augmentation.

Input sequences are provided in FASTA format. For each contig, the software preserves the input order and outputs a predicted taxon in the format TaxonName_TaxonLevel together with a normalized confidence score in the range [0,1].

For a fixed runtime comparison, we sampled 100 fixed 4000-bp test fragments with seed 20260715 and retained the ordered manifest. On one NVIDIA V100, BLASTn required 1.73 s; ViTax-RAG inference reusing the explicitly retained 11-column file blast_100.tsv required 361.65 s, giving 363.38 s end-to-end, while the matched ViTax-BiHyena retrieval-off run required 94.82 s on the identical input. Peak CPU resident memory was 0.130, 2.115, and 2.043 GiB for BLASTn, ViTax-RAG, and ViTax-BiHyena, respectively; peak PyTorch tensor allocation was 10.803 GiB for ViTax-RAG and 0.299 GiB for ViTax-BiHyena. The BLASTn and neural timings are not normalized throughput comparisons because the components use different CPU/GPU paths and output scopes. Software and database provenance is provided in Table S14, available at supplementary material  *Bioinformatics Advances* online, and the complete runtime comparison is provided in Table S15, available at supplementary material  *Bioinformatics Advances* online. Exact inputs, commands, versions, and raw resource logs are provided in the Supplementary Material, available at supplementary material  *Bioinformatics Advances* online.

## 6 Discussion

The continued growth of viral metagenomic data has intensified the need for reliable taxonomic classification, particularly for short and divergent contigs with limited reference support. ViTax-RAG combines BLASTn-derived homology evidence with learned sequence representations and confidence-aware hierarchical classification for dsDNA viral contigs. At comparable family or established-genus endpoints, ViTax-RAG outperformed current viral taxonomy tools, including geNomad, PhaGCN_Cluster, and vConTACT3, on the fixed held-out test, and database-overlap-free subsets retained the same ordering. ViTax-RAG also achieved higher exact-genus accuracy than the supported PhaGCN_Cluster and vConTACT3 outputs across controlled fragment-length and within-genus distribution-shift evaluations, with the clearest advantages for short contigs. Retrieval-negative inputs retained partial sequence-only capability but remained less accurate than hit-positive inputs. On GOV 2.0, the complete pipeline processed 1 95 728 contigs, and 87.2% of predictions terminated at class.

These characteristics could support applications in viral surveillance, public-database triage, low-biomass metagenomics, and degraded samples, where short contigs may be the only actionable sequence evidence. For ecological analyses, ViTax-RAG assignments can annotate dereplicated viral populations or viral operational taxonomic units (vOTUs) after read mapping and abundance normalization; raw fragment incidence should not be interpreted as richness, and class-level assignments should not be overinterpreted as fine-scale community structure.

## Supplementary Material

vbag247_Supplementary_Data

## Data Availability

Detailed algorithms and evaluation procedures are provided in the Supplementary Information. The source code is available from the ViTax-RAG software repository, and ViTax-RAG is distributed through PyPI (version 0.2.1).
