# Supplementary material for: ViTax-RAG: a retrieval-augmented language modeling tool for viral contig taxonomic classification
Source: Bioinform Adv. 2026 Aug 24;6(1):vbag247. doi: 10.1093/bioadv/vbag247 (PMC13615708; doi:10.1093/bioadv/vbag247)
Supplement: Supplementary file 1 [file vbag247_supplementary_data.pdf]

Supplementary Information
ViTax-RAG: A Retrieval-Augmented Language Modeling Tool for Viral Contig Taxonomic Classification

Feng Zhou, Lan Cao, Yushuang He, Jiaxing Bai, and Ying Wang*

National Institute for Data Science in Health and Medicine, State Key Laboratory of Mariculture Breeding, Department of Automation, Xiamen Key Laboratory of Big Data Intelligent Analysis and Decision, Xiamen University, Xiamen, Fujian, China

*Corresponding author: wangying@xmu.edu.cn

6 August 2026

Contents

1 Experimental and parameter provenance **1**

1.1 Candidate-score normalization and ranking 2

2 Rank-aware complete-test evaluation **3**

3 Short-fragment attribution and hit/no-hit analysis **4**

4 Class imbalance **8**

5 OOD benchmark **8**

5.1 Within-genus OOD split 8

6 External database overlap and current-tool comparison **12**

7 GOV 2.0 terminal-rank analysis **14**

8 Software versions, commands, and computational resources **14**

9 Evidence limitations **16**

References **18**

1 Experimental and parameter provenance

Complete-genome rank-aware results were computed from accession-level predictions, whereas fragment analyses used the corresponding fixed fragment manifests. Taxonomic reporting follows ICTV VMR MSL39 v1, release file dated 12 September 2024. The train and test accession sets are disjoint. External-tool databases were audited separately for exact test-accession overlap because their release dates postdate the ViTax-RAG split.

1.1 Candidate-score normalization and ranking

Every query is searched with BLASTn [3]. For a query q, H_q denotes only the candidate hits returned for that query (at most 500 target sequences); it does not include hits from other queries or from an encoder/inference batch. For candidate $i \in H_q$, let E_i, B_i, and L_i denote its E-value, bit score, and alignment length, respectively. The non-negative transformed E-value significance and the per-query maxima are

$$X_i = \max\left\{0, -\log_{10}\left(E_i + 10^{-200}\right)\right\}, \qquad X_q^{\max} = \max_{j \in H_q} X_j, \quad B_q^{\max} = \max_{j \in H_q} B_j, \quad L_q^{\max} = \max_{j \in H_q} L_j.$$

The normalized features are

$$\begin{aligned} \widetilde{I}_i &= \text{pident}_i/100, & \widetilde{C}_i &= \text{qcovs}_i/100, \\ \widetilde{B}_i &= \begin{cases} B_i/B_q^{\max}, & B_q^{\max} > 0, \\ 0, & B_q^{\max} = 0, \end{cases} & \widetilde{E}_i &= \begin{cases} X_i/X_q^{\max}, & X_q^{\max} > 0, \\ 0, & X_q^{\max} = 0, \end{cases} \\ \widetilde{L}_i &= \begin{cases} L_i/L_q^{\max}, & L_q^{\max} > 0, \\ 0, & L_q^{\max} = 0. \end{cases} \end{aligned} \tag{1}$$

Thus, normalization is performed independently for each query using maximum normalization, not min–max normalization. If H_q contains one candidate, every positive maximum-normalized feature equals 1. If all candidates have the same positive alignment length, $\widetilde{L}_i = 1$ for every candidate. A zero maximum gives a normalized value of 0 by definition, avoiding division by zero. When $H_q = \emptyset$, candidate scoring is skipped and the unmodified query follows the no-hit path.

Identity, query coverage, bit score, transformed E-value significance, and alignment length are assigned exponents 0.6, 0.5, 0.5, 0.4, and 0.3, respectively, in the main-text multi-feature score. Gap-opening and mismatch penalties and the 0.5% deterministic near-tie rule are applied before the candidate with the highest composite score S_i^* is selected. These settings and the taxonomy confidence threshold $\theta = 0.6$ were selected using a held-out development split drawn only from the training partition, then locked before all final test and OOD evaluations.

For a hit-positive query, the complete substring `subject[send:]` from the candidate with the highest composite score S_i^*, in stored FASTA orientation, is concatenated to the query. If a reported BLAST subject cannot be located in the indexed reference FASTA collection, the pipeline raises a data-integrity error; the record is not silently treated as a no-hit query. The augmented length is not capped, and there is no query-length-specific retrieval branch. Windowing follows the released historical `split_string4` rule on both forward and reverse-complement strands. Sequences of at least 2,000 bp produce complete 2,000-bp windows beginning every 400 bp while a full window remains, without an additional right-aligned terminal window. A sequence shorter than 2,000 bp is retained once as a variable-length short window; it is not discarded, repeated, or padded to a fixed 2,000-bp length. No-hit queries remain unchanged and proceed through the same Bi-Hyena encoder, K-means index, taxonomy tree, and windowing path as ViTax-RAG; operationally, this is the ViTax-BiHyena retrieval-off path rather than the original ViTax model. Thus 500–1,500-bp no-hit queries remain valid model inputs. Tokenization uses one nucleotide per token and no inserted special tokens. If unequal lengths occur in one batch, `[PAD]` (token ID 4) is added on the left only to the batch maximum, giving an input tensor of shape $B \times L_{\max}$ with $L_{\max} \leq 2{,}000$. In the released per-record inference path, a short sequence forms a one-item $1 \times L$ batch and is therefore not padded. The released inference code passes only token IDs to Bi-Hyena; the tokenizer-generated attention mask is not supplied and no `[MASK]` token is inserted. The same candidate-selection, augmentation, and windowing procedure is used across the ID and OOD evaluations.

Model inference uses NumPy/PyTorch seed 0, deterministic cuDNN behavior, and PyTorch evaluation mode, which disables the 0.1 embedding-dropout layer used during training. All reported ViTax-RAG tables use this deterministic configuration. The OOD checkpoint loads all 118 parameters strictly, although its paired K-means object carries the environment qualification described below.

The dataset was constructed from the 2023 NCBI Virus RefSeq collection, reconciled to ICTV taxonomy, restricted to complete dsDNA viral genomes with usable genus labels, and limited to genera represented by at least two genomes under the taxonomy used to construct the split. Genome-type eligibility and usable genus labels were determined from the RefSeq records and taxonomy used at dataset construction; ICTV VMR MSL39 v1 was applied later only to harmonize current taxon names for reporting. A 6:4 within-genus genome split was performed before 2,000-bp/400-bp fragmentation, with at least one test genome retained for small genera. The formal dataset contains 3,977 complete dsDNA genomes: 2,462 training genomes and 1,515 test genomes. VMR MSL39 provides current taxonomy rows for 2,448 training and 1,505 test entries; this later reconciliation does not redefine the original genome-type eligibility

criterion. The formal partitions jointly span 638 current genera, 72 families, 23 orders, and 12 classes under MSL39. Differences from older taxonomy releases reflect MSL39 renaming, merging, and splitting. Reference-proximal and fragment metrics use only labels present in VMR MSL39. Caudoviricetes account for 73.2% of training genomes.

The formal partition counts and current taxonomic breadth are summarized in Supplementary Tables S1 and S2, respectively.

Table S1: Final formal dataset composition under the source-selection criteria and current VMR MSL39 taxonomic reconciliation.

Characteristic	Training	Test
Accessions	2,462	1,515
Complete dsDNA genomes by selection criterion	2,462	1,515
Official VMR matches	2,448	1,505
Matched species	2,225	1,374
Singleton species	2,186	1,352
Matched genera	632	629
Singleton genera	264	421
Median genomes per genus	2	1
Maximum genomes per genus	111	73
Caudoviricetes genomes	1,803	1,114

Table S2: Current taxonomic breadth under ICTV VMR MSL39 v1.

Scope	VMR-matched accessions	Classes	Orders	Families	Genera
Training	2,448	12	23	72	632
Formal test	1,505	12	23	72	629
Training ∪ formal test	3,953	12	23	72	638

The full accession-level VMR audit and per-species/per-genus counts are provided as machine-readable Supplementary Data. All 12 test classes, 23 orders, and 72 families occur in training; 623/629 current test genera occur in training. The six current test-only names are *Baihuvirus*, *Beetrevirus*, *Guelphvirus*, *Jedunavirus*, *Otagovirus*, and *Pamexvirus*. They arise from applying MSL39 to the fixed accession split; no accession was moved between partitions. The complete split is consequently treated as a reference-proximal known-taxon test rather than a taxonomically disjoint benchmark.

2 Rank-aware complete-test evaluation

ViTax denotes the original published baseline with a unidirectional Hyena encoder. The complete-test table below compares ViTax-RAG with this original model rerun on the same fixed inputs and rescored against the same MSL39 truth. *ViTax-BiHyena* denotes the matched bidirectional sequence-only control: it uses the same checkpoint and encoder configuration, K-means model, taxonomy tree and index, windowing, and confidence threshold as ViTax-RAG, but disables retrieval (`--augment false`); it is used in the retrieval-attribution analyses below rather than as the comparator in the complete-test table.

Precision is $TP/(TP + FP)$, recall is TP/N_{truth}, and F1 is their harmonic mean. Metrics at a rank are restricted to inputs having VMR truth at that rank. N_{truth} is therefore calculated independently at each rank from labels explicitly present in ICTV VMR MSL39 v1; missing ancestral ranks are not imputed. Its non-monotonic values reflect unassigned rank fields rather than a hierarchy-mapping error: 573 genomes have VMR family and genus labels but a blank order field, another 650 have a VMR genus label but blank family and order fields, and 41 have a VMR class label but a blank phylum field. An unclassified prediction or one terminating above the evaluated rank remains in the recall denominator as an unassigned/false-negative case but does not enter the precision denominator. A prediction emitted below the evaluated rank is projected to its lineage ancestor before comparison. In main-text Table 1, N assigned counts truth-evaluable inputs with a lineage prediction at the rank; lineage assignment coverage divides that count by all 1,515 inputs. Any-rank resolution is the fraction of all inputs receiving any non-root label. These measures

are deliberately separated from the terminal-rank distribution in Table S3, which reports the truth-independent deepest rank emitted for every input, including unclassified inputs. Here, `unclassified` means that the taxonomy tree abstained from emitting a non-root known-taxon label; it is not a candidate novel-taxon prediction and does not cluster unclassified sequences into candidate groups.

Operationally, TP is an exact single-label match, $TP + FP = N_{\text{assigned}}$, and therefore precision is TP/N_{assigned}. Pipe-delimited candidate sets count as assignments for lineage coverage but not as exact-label true positives. This same count-based definition is used for every method and rank.

3 Short-fragment attribution and hit/no-hit analysis

An accession-balanced in-silico fragment set was evaluated at 500, 1,000, 1,500, 2,000, 3,000, and 4,000 bp to quantify rank-specific performance and terminal backoff. In this diagnostic, genus precision, recall, and F1 are rank-aware metrics, whereas terminal-rank fractions are truth-independent proportions of all diagnostic inputs at a given length; neither quantity is the all-contig exact-genus accuracy reported in the main text. This diagnostic isolates sequence length but does not reproduce assembly errors or the selection process that creates genuinely short metagenomic contigs. The rank-aware comparison and terminal-rank distributions are reported in Supplementary Tables S4 and S5, respectively; the all-contig accuracy ablation and OOD hit/no-hit analysis are reported in the main text.

Table S3: Deepest rank emitted on the complete test. Values are percentages except N.

Method	N	Unclassified (%)	Class (%)	Order (%)	Family (%)	Genus (%)
ViTax-RAG	1515	1.8	1.3	0.1	3.7	93.2
ViTax	1515	2.6	2.4	0.0	3.8	91.2
geNomad v1.12.0	1515	7.3	45.9	0.4	46.0	0.0
PhaGCN_Cluster	1515	16.0	19.5	0.0	49.3	15.2
vConTACT3 v3.1.6	1515	4.5	0.0	0.0	0.0	95.5

Table S4: Accession-balanced ViTax-RAG versus ViTax-BiHyena retrieval-only comparison. Any-rank resolution and genus metrics are percentages.

Method	Length	N	Any-rank resolution (%)	Genus P	Genus R	Genus F1	Lineage accuracy
ViTax-BiHyena	500	1515	44.1	2.2	0.1	0.3	71.8
ViTax-RAG	500	1515	87.0	94.5	74.9	83.6	90.9
ViTax-BiHyena	1000	1515	44.8	56.8	9.8	16.7	75.9
ViTax-RAG	1000	1515	89.2	94.5	80.7	87.1	92.7
ViTax-BiHyena	1500	1515	79.7	87.6	64.1	74.0	88.1
ViTax-RAG	1500	1515	93.4	93.6	86.6	90.0	93.4
ViTax-BiHyena	2000	1508	92.1	91.2	81.6	86.1	91.1
ViTax-RAG	2000	1508	96.8	93.5	89.1	91.2	93.3
ViTax-BiHyena	3000	1476	95.7	93.1	84.3	88.5	93.2
ViTax-RAG	3000	1476	98.0	94.1	90.0	92.0	94.0
ViTax-BiHyena	4000	1458	95.3	96.7	84.4	90.1	96.6
ViTax-RAG	4000	1458	97.9	96.3	91.0	93.6	96.1

Table S5: Deepest emitted rank for ViTax-RAG and ViTax-BiHyena at each controlled fragment length. Percentages use all fixed-test inputs at the stated length, including unclassified inputs; genus percentages are terminal output proportions, not accuracy.

Method	Length	N	Unclassified (%)	Class (%)	Order (%)	Family (%)	Genus (%)
ViTax-BiHyena	500	1,515	55.9	37.7	0.1	0.4	5.9
ViTax-RAG	500	1,515	13.0	7.7	0.0	0.1	79.2
ViTax-BiHyena	1000	1,515	55.2	26.9	0.0	0.8	17.1
ViTax-RAG	1000	1,515	10.8	3.6	0.1	0.1	85.5
ViTax-BiHyena	1500	1,515	20.3	4.7	0.1	1.6	73.3
ViTax-RAG	1500	1,515	6.6	0.3	0.1	0.5	92.5
ViTax-BiHyena	2000	1,508	7.9	1.6	0.0	1.1	89.5
ViTax-RAG	2000	1,508	3.2	0.6	0.0	1.0	95.2
ViTax-BiHyena	3000	1,476	4.3	2.7	0.0	2.4	90.6
ViTax-RAG	3000	1,476	2.0	0.4	0.0	1.9	95.7
ViTax-BiHyena	4000	1,458	4.7	3.4	0.1	4.3	87.4
ViTax-RAG	4000	1,458	2.1	0.3	0.1	2.9	94.6

At 500 bp, ViTax-RAG achieved 94.5% genus precision, 74.9% recall, and 83.6% F1, whereas ViTax-BiHyena achieved 2.2%, 0.1%, and 0.3%. ViTax-RAG terminated at genus for 79.2% of inputs, compared with 5.9% for ViTax-BiHyena. Predictions terminating at class or family remain false negatives in genus recall and receive no genus-level credit.

Separately, the original ViTax checkpoint and parameter setting were trained and evaluated using 4,000-bp inputs represented internally as overlapping 2,000-bp windows, whereas its historical 500-bp result applied that setting without retraining or short-input retuning. The published ViTax evaluation began at 4,000 bp, where genus accuracy was 0.923 on RefSeq and 0.648 on the OOD split (He et al., 2025; doi:10.1093/bib/bbaf041). The original ViTax accuracy of 0.008 at 500 bp therefore reflects a strong length-distribution shift and is not ViTax's OOD accuracy at its intended input length. This historical ViTax result is distinct from the matched ViTax-BiHyena retrieval-off results above. Kraken2 v2.1.3 and CAT_pack v6.0 used databases constructed from the same training sequence collections as the corresponding ViTax-RAG experiments: the ICTV/RefSeq training set for the ID evaluation and the OOD training genomes for the OOD evaluation. Their training-set-matched accuracy values are included in main-text Table 2; this endpoint is reported separately from the VMR genus-F1 analysis.

The fragment-length classification comparison is reported in main-text Table 2 and includes ViTax-RAG, Kraken2, CAT, geNomad, PhaGCN_Cluster, and vConTACT3. Panel A retains the original RefSeq ViTax column as a historical unidirectional baseline. The OOD ViTax-RAG values are the count-weighted hit/no-hit results, and the OOD Kraken2 values use the strict database rebuilt exclusively from the 1,466 OOD training accessions; the RefSeq values and OOD CAT values are retained from the prior benchmark. The overlapping RefSeq ViTax-RAG and ViTax entries use the same endpoint and values as the main-text ID ablation table; in both locations, ViTax-RAG accuracy at 4,000 bp is 0.947. The endpoint is established-genus classification accuracy, calculated as the number of fragments recovering the truth genus divided by all fragments at the stated length; missing and unclassified outputs remain in the denominator. This sequence-classification endpoint is distinct from the rank-specific precision, recall, and F1 definitions used for the complete-genome analysis above.

For main-text Table 2A and 2B, geNomad v1.12.0/database v1.9 was run with `--lenient-taxonomy` to expose its available genus assignments; the complete-genome per-rank comparison used geNomad's standard conservative mode. The PhaGCN column in the original working table corresponds to the genus-capable PhaGCN_Cluster implementation (official commit `8da942a`, bundled MSL39 database). Inputs shorter than its documented 1,700-bp minimum are unsupported and are shown as dashes. For vConTACT3 v3.1.6/database v230, a pipe-delimited candidate set is counted as correct when the truth genus occurs anywhere in the set; its entries therefore represent candidate-set hit accuracy rather than the stricter exact-single-label P/R/F1 used in main-text Table 1. geNomad, PhaGCN_Cluster, and vConTACT3 use their current official databases rather than databases built from the ViTax-RAG training partitions, so the tables are descriptive current-tool comparisons rather than leakage-free generalization estimates. The displayed values are also provided as `table_s_fragment_accuracy_all_methods.csv`.

To diagnose the low all-fragment accuracies without changing their prespecified denominator, genus-call coverage was calculated as $N_{\mathrm{called}}/N_{\mathrm{fragments}}$, and conditional correctness as $N_{\mathrm{correct}}/N_{\mathrm{called}}$. These quantities explain whether a method primarily abstained at genus rank or emitted a genus/candidate label that did not match the established truth; they are not alternative headline metrics.

ID (RefSeq) results.

- **geNomad:** genus-call coverage was 21.5–35.8% across 500–4,000 bp, whereas 74.9–77.4% of called fragments matched the truth genus. Its 0.162360–0.277235 all-fragment accuracy is therefore mainly coverage-limited. The lenient option permits but does not force a genus, and short fragments frequently contain too few complete genes or informative taxonomy markers for fine-rank assignment. Current-database versus benchmark-taxonomy differences can additionally contribute to mismatches among called fragments.

- **PhaGCN_Cluster:** 500–1,500-bp inputs are outside the documented operating range. At 2,000–4,000 bp, genus-call coverage was only 5.4–8.6%, although 75.6–79.1% of those calls were correct; all-fragment accuracy was consequently 0.041186–0.066806. The method depends on predicted proteins, gene-sharing edges, and graph connectivity, and an isolated short fragment often provides too few genes to connect to a genus-resolved reference cluster.

- **vConTACT3:** genus/candidate-call coverage increased from 48.7% to 93.5%, but only 0.006–0.186% of called fragments contained the exact truth genus. vConTACT3 frequently emits candidate identifiers such as `novel_genus_*`; these are useful gene-sharing cluster labels but are not equal to established ICTV genus names. Fragmentation also reduces shared-gene context and can place different pieces of one genome into different candidate clusters.

Within-genus OOD results.

- **geNomad:** genus-call coverage was 21.5–38.6%, while conditional correctness remained 75.7–79.6%. Its 0.170892–0.292131 accuracy is therefore again primarily coverage-limited rather than an OOD-specific collapse. Because the current official database was used instead of a database restricted to the OOD training genomes, these values are not a leakage-free independent-generalization estimate.
- **PhaGCN_Cluster:** at 2,000–4,000 bp, genus-call coverage was 8.0–11.5% and conditional correctness was 73.1–83.7%, yielding 0.058104–0.085328 all-fragment accuracy. The short-fragment protein/graph-connectivity limitation again dominates. Its current database contains 435/494 (88.1%) OOD test accessions, so the low value cannot be attributed solely to biological novelty; most fragments still lack a genus-resolved established label.
- **vConTACT3:** a genus or candidate-genus output was present for 41.4–81.7% of fragments, but conditional exact-truth recovery was only 0–0.305%, yielding 0–0.002489 all-fragment accuracy. Candidate-cluster names do not generally equal ICTV truth labels, and short/divergent fragments provide less shared-protein evidence. The v230 database contains 332/494 (67.2%) OOD test accessions, so this result measures exact known-label compatibility under the current database rather than a clean novelty benchmark.

The low values therefore reflect two different mechanisms: geNomad and PhaGCN_Cluster primarily lose score through genus-level abstention or unresolved output, whereas vConTACT3 primarily loses score because its candidate-cluster identities are not equivalent to the established-genus labels used by this endpoint. This does not imply poor viral detection or poor candidate-taxon organization.

4 Class imbalance

Class counts, pooled class stratification, and per-class performance are reported in Supplementary Tables S6, S7, and S8, respectively.

The substantially lower non-Caudoviricetes F1 limits extrapolation from the pooled result and is consistent with the 73.2% Caudoviricetes share in training. Among classes represented by at least 20 final-test genomes, genus F1 ranged from 63.3% for Papovaviricetes to 100% for Pokkesviricetes, further demonstrating that the pooled score is not uniformly representative. ViTax-RAG family F1 was 97.7%, compared with 86.7% for the supporting family-level PhaGCN2.0 result, 87.2% for PhaGCN_Cluster, and 58.4% for vConTACT3. Across the complete set, ViTax-RAG correctly recovered 565 distinct truth genera, compared with 20 for PhaGCN_Cluster and 439 for vConTACT3. These counts evaluate exact recovery of established ICTV genus labels and do not treat candidate novel clusters as known genera.

5 OOD benchmark

5.1 Within-genus OOD split

The split contains 1,466 OOD training accessions and 494 OOD test accessions (1,960 total). Both subsets contain the same 143 genera. It was constructed to hold out a genomically separated cluster within each genus while preserving genus truth for supervised evaluation. It therefore tests generalization to shifted members of known genera, not recognition of unseen genera. The construction command was:

```
./dashing_s256 dist --containment-index -k21 \
  -O GENUS_OUTPUT.tsv -o fsizes.txt \
  -Q GENUS_FASTA -F GENUS_FASTA
```

Dashing version v1.0.2-4-g0635 [1] was used. No explicit `-S` option was passed, so the HyperLogLog sketch used the documented default size of 1,024 bytes. The containment index estimates the directional fraction of one genome's 21-mer set contained in another genome's 21-mer set. The data-preparation procedure calculated each genome's row-mean containment against the other genomes in its genus, removed genomes with a mean above 0.3 to reduce highly redundant representatives, and applied two-cluster spectral clustering to the remaining within-genus containment matrix (precomputed affinity; random seed 42). Thus 0.3 is a cutoff on mean directional containment, not a Jaccard

Table S6: Genome counts across every current VMR MSL39 class label represented in the retained training and final-test metadata. “Unassigned” denotes entries without a current MSL39 class mapping. These reporting labels do not redefine the complete-dsDNA source-selection criterion.

Class	Training	Final test
Caudoviricetes	1803	1114
Unassigned	196	128
Papovaviricetes	155	90
Herviviricetes	72	40
Naldaviricetes	64	41
Tectiliviricetes	60	37
Megaviricetes	43	23
Pokkesviricetes	38	24
Tokiviricetes	16	9
Huolimaviricetes	7	4
Laserviricetes	6	3
Maveriviricetes	1	1
Revtraviricetes	1	1

or pairwise identity threshold. Because it depends on asymmetric containment, genome length, and averaging across multiple genomes, no ANI or Mash-ANI equivalent is assigned.

For leakage control, the BLAST database was constructed exclusively from the 1,466 OOD training genomes, comprising 73,997,559 bp in total. Its full FASTA SHA-256 is provided in the provenance record and reproduction guide. Train/test accession overlap and canonical forward/reverse-complement exact-sequence overlap were zero, and no test accession appeared as its own top subject. Same-genus training references are part of the benchmark definition.

The workflow below uses descriptive publication-facing filenames; the reproduction guide provides the corresponding archived paths and file hashes.

```
python experiments/prepare_ood_clean_rerun.py \
  --train-xlsx OOD_TRAINING_ACCESSIONS.xlsx \
  --test-xlsx OOD_TEST_ACCESSIONS.xlsx \
  --sequence-dir data/sequence --out-dir OOD_RUN/prepared \
  --lengths 500 1000 1500 2000 3000 4000
makeblastdb -in OOD_RUN/prepared/OOD_TRAINING_GENOMES.fasta \
  -dbtype nucl -out OOD_RUN/blastdb/OOD_TRAINING_GENOMES
blastn -query TEST_FRAGMENTS.fasta \
  -db OOD_RUN/blastdb/OOD_TRAINING_GENOMES \
  -task megablast -evalue 10 -max_target_seqs 500 \
  -outfmt "6 qseqid sseqid sstart send pident length \
  mismatch gapopen bitscore evalue qcovs" \
  -out OOD_HITS.tsv
```

Table S7: ViTax-RAG genus metrics stratified by Caudoviricetes versus all other classes.

Method	Stratum	N truth	N genus	Terminal genus fraction (%)	Precision (%)	Recall (%)	F1 (%)
ViTax-RAG	All	1505	1402	92.5	97.9	91.2	94.5
ViTax-RAG	Caudoviricetes	1114	1091	97.9	99.2	97.1	98.1
ViTax-RAG	Non-Caudoviricetes	273	201	73.6	95.0	70.0	80.6

Table S8: ViTax-RAG genus performance for each assigned VMR class in the final test. Classes represented by only one to four genomes have very high uncertainty and should not be interpreted comparatively.

Class	N truth	N genus	Precision (%)	Recall (%)	F1 (%)
Caudoviricetes	1114	1091	99.2	97.1	98.1
Papovaviricetes	90	49	89.8	48.9	63.3
Naldaviricetes	41	36	100.0	87.8	93.5
Herviviricetes	40	28	96.4	67.5	79.4
Tectiliviricetes	37	30	93.3	75.7	83.6
Pokkesviricetes	24	24	100.0	100.0	100.0
Megaviricetes	23	21	95.2	87.0	90.9
Tokiviricetes	9	7	85.7	66.7	75.0
Huolimaviricetes	4	3	100.0	75.0	85.7
Laserviricetes	3	3	100.0	100.0	100.0
Maveriviricetes	1	0	0.0	0.0	0.0
Revtraviricetes	1	0	0.0	0.0	0.0

```
python experiments/evaluate_ood_clean_rerun.py --root OOD_RUN
```

The six non-overlapping fragment sets contain 69,143, 34,459, 22,882, 17,090, 11,315, and 8,438 fragments at 500, 1000, 1500, 2000, 3000, and 4000 bp, respectively. When a genome is shorter than the nominal length, the fragmenter retains it once as its complete sequence; every nominal length therefore represents all 494 test accessions. ViTax-RAG, ViTax-BiHyena, and BLAST-only top-hit label transfer were evaluated against the same manifests and genus truth. ViTax-BiHyena uses the identical OOD-specific checkpoint, encoder, K-means model, taxonomy tree, and index as ViTax-RAG with the augmentation step omitted. The top-hit control retains the first-ranked subject accession and emits its OOD training-set genus; it does not compute an LCA across hits, and no-hit fragments remain unclassified. The resulting BLAST hit rates, BLAST-only top-hit accuracies, and ViTax-RAG hit/no-hit accuracies are reported in main-text Table 3B.

PhaGCN_Cluster (official commit `8da942a`, bundled MSL39 database) and vConTACT3 v3.1.6 (v230 database) were additionally run on the same OOD test-fragment FASTA files. These are current-database comparisons, not reference systems constructed from the OOD training genomes. Exact-accession audit found 435/494 (88.1%) OOD test accessions in the PhaGCN_Cluster database and 332/494 (67.2%) in vConTACT3; method-specific clean subsets therefore contain 59 and 162 accessions, respectively. To make each clean-subset comparison paired, ViTax-RAG was re-evaluated on exactly the same fragments from those accessions. PhaGCN_Cluster was run at 2000–4000 bp; shorter inputs are below its documented default 1700-bp minimum and are shown as unsupported. vConTACT3 was run at all six lengths. Supplementary Table S9 scores exact recovery of the established ICTV genus. Candidate novel clusters remain biologically useful outputs but are not counted as correct established-genus labels.

Table S9: Exact established-genus F1 (%) for current external tools on the Dashing within-genus OOD fragments. "All" uses all 494 OOD test accessions; PhaGCN-clean and vContACT-clean are the method-specific exact-accession-overlap-free subsets with 59 and 162 accessions. Dashes indicate inputs below the PhaGCN_Cluster default minimum length, not zero performance. These tools use their current official databases rather than a database constructed from the OOD training genomes.

Length (bp)	ViTax-RAG all	PhaGCN_Cluster all	ViTax-RAG PhaGCN-clean	PhaGCN_Cluster PhaGCN-clean	vConTACT3 all	ViTax-RAG vConTACT-clean	vConTACT3 vConTACT-clean
500	50.61	–	19.92	–	0.00	17.28	0.00
1000	59.41	–	26.55	–	0.01	28.26	0.00
1500	64.30	–	31.96	–	0.02	35.41	0.00
2000	69.75	10.76	36.39	2.60	0.03	45.82	0.02
3000	72.04	14.44	38.10	3.26	0.10	49.82	0.05
4000	73.76	15.30	39.18	3.25	0.27	52.22	0.03

On all fragments, PhaGCN_Cluster genus F1 was 10.8–15.3% at 2000–4000 bp and vConTACT3 genus F1 was 0–0.27% across all six lengths, compared with 50.6–73.8% for ViTax-RAG. On the paired clean subsets, ViTax-RAG/PhaGCN_Cluster F1 was 36.4%/2.6%, 38.1%/3.3%, and 39.2%/3.3% at 2000, 3000, and 4000 bp, while ViTax-RAG/vConTACT3 ranged from 17.3–52.2% versus 0–0.05%. The lower clean-subset ViTax-RAG values relative to its all-fragment values show that these accessions are unusually difficult; the paired comparison nevertheless retains a large exact-label gap. This analysis supports ViTax-RAG's known-genus recovery on short fragments, not superiority for candidate-cluster discovery or a claim that the external tools' reference databases were constructed exclusively from the OOD training genomes.

The F1 values in the preceding paragraph are rank-aware, whereas the hit/no-hit values in main-text Table 3B are exact-genus accuracies using every fragment in the corresponding stratum as the denominator; these endpoints are not numerically interchangeable. The OOD hit rate increased from 0.5431 at 500 bp to 0.6925 at 4,000 bp. ViTax-RAG accuracy was 0.8370–0.8836 in hit-positive contigs, whereas no-hit accuracy declined from 0.3343 at 500 bp to 0.2328 at 4,000 bp. Thus, the retrieval-negative sequence-only path retained limited but non-zero classification capability, but performance remained substantially lower than for hit-positive inputs. Because a no-hit query is not augmented, that fallback is the ViTax-BiHyena path, not the original ViTax model. The all-fragment values are the count-weighted averages of the two strata; for example, the 500-bp result is $(37,555 \times 0.8836 + 31,588 \times 0.3343)/69,143 = 0.6327$, reported as 0.633.

The matched fragment-level established-genus comparison is reported in main-text Table 2B. ViTax-RAG accuracy was 63.3/64.7/65.5/66.6/64.9/67.3% across the six lengths. The corresponding strict training-set-matched Kraken2 accuracies were 50.0/55.7/58.5/60.5/62.8/64.6%, and CAT accuracies were 50.2/52.7/54.5/55.6/56.9/57.4%. ViTax-RAG was higher than both matched baselines at every length.

The `dash2.pth` checkpoint loads all 118 parameter tensors strictly, including its learned position embeddings and embedding LayerNorm. The paired K-means pickle reports scikit-learn 0.23.2, whereas evaluation under 1.5.2 raises an `InconsistentVersionWarning`; a 100-input smoke comparison reproduces 98/100 reference genus labels but not identical confidence values. The OOD results are therefore environment-qualified rather than bit-exact.

6 External database overlap and current-tool comparison

Exact database overlap and overlap-free subset performance are reported in Supplementary Tables S10 and S11, respectively.

Table S10: Exact accession overlap between the 1,515-genome final test and downloaded current external databases.

Database	N reference	N overlap	Overlap (%)	N test clean
PhaGCN2.3/PhaGCN_Cluster VMR_MSL39_v1	7279	1079	71.2	436
vConTACT3 RefSeq v230 prokaryotes	4589	1124	74.2	391
Combined PhaGCN/vConTACT3 exact-accession audit	9240	1360	89.8	155

Table S11: Selected family/genus metrics on external-database accession-overlap-free subsets. The method-specific clean subsets contain 436 PhaGCN-clean and 391 vConTACT3-clean genomes; the joint clean subset contains 155 genomes.

Subset	Method	Rank	N	N_{truth}	N_{assigned}	Precision (%)	Recall (%)	F1 (%)
combined_external_accession_overlap_free	ViTax-RAG	Family	155	146	142	97.9	95.2	96.5
combined_external_accession_overlap_free	ViTax-RAG	Genus	155	148	135	91.9	83.8	87.6
combined_external_accession_overlap_free	ViTax-BiHyena	Family	155	146	139	97.8	93.2	95.4
combined_external_accession_overlap_free	ViTax-BiHyena	Genus	155	148	132	93.2	83.1	87.9
combined_external_accession_overlap_free	geNomad v1.12.0	Family	155	146	37	100.0	25.3	40.4
combined_external_accession_overlap_free	geNomad v1.12.0	Genus	155	148	–	–	–	–
combined_external_accession_overlap_free	PhaGCN_Cluster	Family	155	146	8	75.0	4.1	7.8
combined_external_accession_overlap_free	PhaGCN_Cluster	Genus	155	148	1	0.0	0.0	0.0
phagcn_accession_overlap_free	PhaGCN_Cluster	Family	436	189	11	54.5	3.2	6.0
phagcn_accession_overlap_free	PhaGCN_Cluster	Genus	436	426	48	0.0	0.0	0.0
combined_external_accession_overlap_free	vConTACT3 v3.1.6	Family	155	146	85	2.4	1.4	1.7
combined_external_accession_overlap_free	vConTACT3 v3.1.6	Genus	155	148	87	3.4	2.0	2.6
vcontact3_accession_overlap_free	vConTACT3 v3.1.6	Family	391	380	314	5.1	4.2	4.6
vcontact3_accession_overlap_free	vConTACT3 v3.1.6	Genus	391	384	318	2.8	2.3	2.6

Complete-test external-tool values are descriptive because the downloaded databases post-date the ViTax-RAG split and contain many exact accessions. The clean-subset values control for this overlap.

7 GOV 2.0 terminal-rank analysis

The official GOV 2.0 at-least-10-kb/circular FASTA [2] contains 195,728 contigs with a median length of approximately 16.2 kb and a range of 10–505 kb. The complete length distribution is reported in Supplementary Table S12.

Table S12: Sequence-length distribution of the complete GOV 2.0 at-least-10-kb/circular dataset.

Length interval	Number of contigs	Proportion (%)
10–20 kb	125,308	64.0
20–30 kb	35,641	18.2
30–50 kb	25,135	12.8
>50 kb	9,644	4.9
Total	195,728	100.0

The complete ViTax-RAG prediction record contains one terminal rank and confidence score for each of the 195,728 contigs, with no duplicate or missing contigs. Its direct aggregation is reported in Supplementary Table S13.

The complete terminal-rank table was generated from one versioned prediction record using `evaluate_gov2_stratified_baselines.py`. The machine-readable provenance record, including the parser hash and exact ID accounting, is available as `gov2_baselines/gov2_terminal_rank_provenance.json`.

Table S13: Terminal rank of ViTax-RAG predictions for all 195,728 official GOV 2.0 contigs. Each contig contributes once at its deepest emitted rank. These values describe output resolution and hierarchical backoff, not taxonomic accuracy.

Terminal outcome	Number of contigs	Proportion (%)
Unclassified (root)	1,475	0.754
Class	170,676	87.201
Order	2,956	1.510
Family	16,634	8.499
Genus	3,987	2.037
Total	195,728	100.000

The direct table shows that class-level backoff dominates (87.201%), while 8.499% and 2.037% of contigs terminate at family and genus, respectively. Figure S1 shows the composition of the emitted hierarchy but is not used as a substitute for this terminal-rank distribution.

8 Software versions, commands, and computational resources

Software and database provenance is summarized in Supplementary Table S14.

Representative commands were:

```
blastn -query QUERY.fasta -db TRAINING_DB -task megablast \
  -evalue 10 -max_target_seqs 500 -out HITS.tsv \
  -outfmt "6 qseqid sseqid sstart send pident length \
  mismatch gapopen bitscore evalue qcovs"
genomad end-to-end INPUT.fasta OUTPUT GENOMAD_DB --threads 24
python run_Speed_up.py --contigs INPUT.fasta --len 2000 --outpath OUTPUT
vcontact3 run --nucleotide INPUT.fasta --output OUTPUT --db-path DB.json \
  --db-version 230 --threads 48 --reduce-memory
```

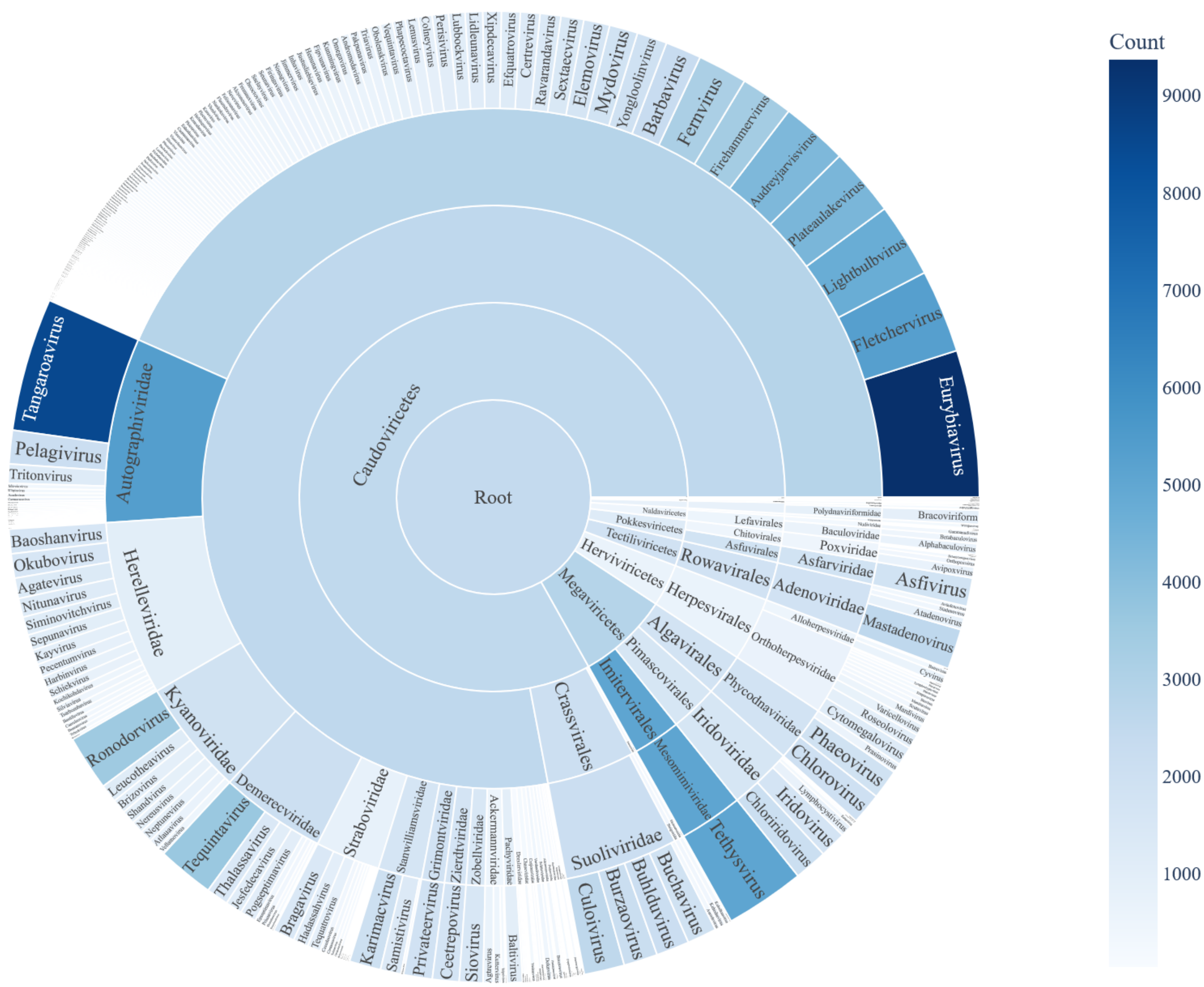

Figure S1: Hierarchical taxonomic composition of GOV 2.0 contigs predicted by ViTax-RAG. The sunburst provides a complementary composition visualization; terminal-rank backoff is reported directly in the accompanying complete-data table, and no taxonomic correctness is inferred without ground truth.

Alt text: Sunburst chart of the taxonomic hierarchy assigned to 195,728 GOV 2.0 contigs. Rings progress outward from the root through successively finer ranks, and darker blue segments indicate larger contig counts. Caudoviricetes occupies most of the inner class ring, with Autographiviridae, Herelleviridae, Kyanoviridae, Demerecviridae, and many smaller branches shown at finer ranks. The chart describes predicted composition; exact terminal-rank counts and backoff proportions are given in Supplementary Table S13.

Table S14: Software and database provenance for the reported experiments.

Component	Version/database	Role
BLAST+	2.12.0+	Nucleotide retrieval
Kraken2	2.1.3 / training-set-matched database	Metagenomic taxonomy baseline
CAT_pack	6.0 / training-set-matched database	Metagenomic taxonomy baseline
Dashing	v1.0.2-4-g0635	Containment split
geNomad	1.12.0 / database 1.9	Current virome comparator
PhaGCN_Cluster	commit `8da942a` / bundled MSL39	Current comparator
vConTACT3	3.1.6 / v230	Candidate-taxon comparator
ViTax-RAG GPU environment	PyTorch 1.12.1 + CUDA 11.3	Deterministic inference
LaTeX	TeX Live 2022 / latexmk 4.76	Document build

The BLAST command used both query strands (the BLASTn default) and applied no additional identity, query-coverage, alignment-length, or bit-score filter after BLAST. Candidate hits were ranked by the main-text multi-feature score and deterministic near-tie rule before selection for augmentation. Kraken2 v2.1.3 and CAT_pack v6.0 used databases constructed from the same training sequence collections as the corresponding ViTax-RAG experiments. Wall-clock logs were not recorded for these runs, so no retrospective Kraken2/CAT runtime is claimed. Current-tool comparisons used the explicitly versioned software and databases listed in Supplementary Table S14.

Runtime and memory were measured on an NVIDIA V100 using the same suffix-augmentation and complete-window rules as the accuracy evaluation. To define a direct and reproducible with/without-retrieval comparison, 100 inputs were sampled with seed 20260715 from the fixed formal 4,000-bp fragment set. The exact input file is `test_fragments_4000_runtime100_seed20260715.fasta`; `runtime100_manifest.csv` records the fixed order, parent accession, length, and per-sequence hash. BLASTn generated the explicitly retained 11-column `blast_100.tsv` file (2,772 rows; 98/100 queries with a hit). ViTax-RAG reused only this file; ViTax-BiHyena used the same 100 FASTA records and artifacts with retrieval disabled. Results are reported in Supplementary Table S15; SHA-256 values for the input, manifest, BLAST output, and predictions are supplied with the raw benchmark logs.

Table S15: Fixed 100-sequence runtime comparison on one NVIDIA V100. Wall time includes model and artifact loading. GPU values are PyTorch peak tensor allocations.

Operation	N	Wall time	Peak CPU RSS (GiB)	GPU tensor peak (GiB)
BLASTn retrieval	100	1.73 s	0.130	–
ViTax-RAG inference using `blast_100.tsv`	100	361.65 s	2.115	10.803
ViTax-BiHyena	100	94.82 s	2.043	0.299
ViTax-RAG end-to-end (BLAST + inference)	100	363.38 s	2.115	10.803

9 Evidence limitations

No independent, accessible dataset of ground-truthed 500-bp–4-kb viral contigs assembled from real samples was available for this study. The short-input evaluation therefore uses controlled in-silico fragments and does not claim independent real-short-contig validation.

The dataset was constructed from the 2023 NCBI Virus RefSeq collection by ICTV reconciliation, restriction to complete dsDNA genomes with usable genus labels, retention of at least two genomes per supervised genus, and a 6:4 within-genus split before fragmentation. It contains 3,977 genomes: 2,462 training genomes and 1,515 test genomes. Under VMR MSL39 v1, the formal training and test partitions jointly span 638 current genera, 72 families, 23 orders, and 12 classes.

No result in this supplement should be interpreted as evidence that ViTax-RAG can name an unseen family or genus. For reference-poor environmental data, candidate-taxon organization by gene-sharing or protein-profile tools addresses a different capability from known-label transfer.

The versioned reproducibility package contains the portable source, `environment.yml`, external-resource layout, installation and example commands, augmentation regression tests, and a stepwise reproduction guide. The fixed

runtime input, manifest, `blast_100.tsv`, predictions, commands, hashes, and raw resource logs are packaged separately as `ViTaxRag_runtime_benchmark_100_20260715.tar.gz`. ViTax-RAG is distributed through PyPI as version 0.2.1; the source snapshot and artifact hashes identify the code and resources used for the reported analyses.

References

[1] D. N. Baker and B. Langmead. Dashing: fast and accurate genomic distances with HyperLogLog. *Genome Biology*, 20(1):265, 2019.

[2] A. C. Gregory, A. A. Zayed, N. Conceição-Neto, B. Temperton, B. Bolduc, A. Alberti, M. Ardyna, K. Arkhipova, M. Carmichael, C. Cruaud, et al. Marine DNA viral macro- and microdiversity from pole to pole. *Cell*, 177(5):1109–1123.e14, 2019.

[3] S. F. Altschul, W. Gish, W. Miller, E. W. Myers, and D. J. Lipman. Basic local alignment search tool. *Journal of Molecular Biology*, 215(3):403–410, 1990.
